# Yield of TB screening in prisons in Tajikistan

**DOI:** 10.5588/ijtldopen.24.0272

**Published:** 2024-08-01

**Authors:** S. Moe, M. Asozoda, A. Aung, Z. Dusmatova, F. Akintore, E. Nshimiyimana, A. Zavqibekov, N. Sitali, W.K. Mulanda, T. Cullip, A. Sinha

**Affiliations:** ^1^ Médecins Sans Frontières, Dushanbe, Tajikistan;; ^2^ Penitentiary Medical Service Department, Ministry of Justice, Dushanbe, Tajikistan;; ^3^ Médecins Sans Frontières (MSF), Berlin, Germany;; ^4^ MSF, London, UK

**Keywords:** active case-finding, prison, tuberculosis screening

## Abstract

**BACKGROUND:**

The rate of TB in prison institutions is estimated to be 23 times higher than in the general population. Limited documentation exists regarding TB screening in Tajikistan's prisons. This study aims to report findings from a TB screening conducted in prison facilities in Tajikistan.

**METHODS:**

A systematic TB screening was conducted between July 2022 and September 2023, following a locally adapted algorithm based on WHO recommendations. The screening yield was calculated as the proportion of confirmed TB cases, with categorical variables compared using a χ^2^ test.

**RESULTS:**

A total of 7,223 screenings were conducted, identifying 31 TB cases, including 17 drug-susceptible TB cases, eight drug-resistant TB cases, and six clinically diagnosed cases. The overall screening yield was 0.43%. Notably, the screening yield was 3.4% among individuals with at least one TB symptom and 0.03% among those without TB symptoms (P < 0.001).

**CONCLUSION:**

The identified rate of TB in these prisons is five times higher than in the general population. Symptomatic individuals had a higher likelihood of TB diagnosis, and using chest X-rays significantly improved screening yield. We recommend increasing the capacity for chest X-ray testing to enhance TB prevention and control within prison settings.

## RÉSUMÉ

**CONTEXTE:**

On estime que le taux de TB dans les établissements pénitentiaires est 23 fois plus élevé que dans la population générale. Il existe peu de documentation sur le dépistage de la TB dans les prisons du Tadjikistan. Cette étude vise à rendre compte des résultats d'un dépistage de la TB mené dans des établissements pénitentiaires au Tadjikistan.

**MÉTHODES:**

Un dépistage systématique de la TB a été réalisé entre juillet 2022 et septembre 2023, selon un algorithme adapté localement et basé sur les recommandations de l'OMS. Le rendement du dépistage a été calculé comme la proportion de cas confirmés de TB, avec des variables catégorielles comparées à l'aide d'un test χ2.

**RÉSULTATS:**

Au total, 7 223 dépistages ont été effectués, permettant d'identifier 31 cas de TB, dont 17 cas de TB sensible aux médicaments, 8 cas de TB résistante aux médicaments et 6 cas diagnostiqués cliniquement. Le rendement global du criblage était de 0,43%. Notamment, le rendement du dépistage était de 3,4% chez les personnes présentant au moins un symptôme de la TB et de 0,03% chez celles ne présentant pas de symptômes de la TB (*P* < 0,001).

**CONCLUSION:**

Le taux de TB identifié dans ces prisons est cinq fois plus élevé que dans la population générale. Les personnes symptomatiques avaient une probabilité plus élevée d'être diagnostiquées comme atteintes de TB, et l'utilisation de radiographies pulmonaires améliorait considérablement le rendement du dépistage. Nous recommandons d'augmenter la capacité de dépistage par radiographie thoracique afin d'améliorer la prévention et le contrôle de la TB en milieu carcéral.

Globally, TB remains a major public health threat, being the second leading cause of death from a single infectious agent in 2022.^1^ The rate of TB can be significantly higher in prisons than in the general population. A systematic review showed that the TB incidence rate is 23 times higher in prisons than in the general population. The study found a substantial difference in the annual incidence of TB in prisons between high-income countries (237.6/100,000 persons) and middle/low-income countries (1,942.8/100,000 persons).^2^

Tajikistan is classified as a high TB burden country,^1^ with an estimated incidence of 78 cases/100,000 population.^3^ The WHO estimated 7,800 new TB cases in 2022; however, only 4,294 cases were reported, implying a 45% treatment initiation gap.^3^ In 2022, 70 new and relapse TB cases were reported from prisons in Tajikistan, representing a case notification rate of 583 cases/100,000 prisoners, higher than the WHO European Region’s average of 463/100,000 prisoners.^4^ The TB relative risk in Tajikistan's prisons is estimated to be 13.5 times higher than in the general population.^4^

The healthcare system in Tajikistan is characterised by centralisation with a primary focus on hospital-based secondary and tertiary care. Efforts have been made to transform the healthcare system towards primary healthcare with support from the WHO and other international organisations.^5^ Tajikistan has 19 correctional facilities, including 13 male prisons, one female prison, one juvenile prison, and four pre-trial detention centres. In 2020, the country had 13,300 detainees. Challenges such as overcrowding and limited resources impact the overall well-being of incarcerated individuals.^6^

The Republican Centre for TB Control (RCTC), under the Ministry of Health and Social Protection of Population (MoH) in Tajikistan, is responsible for TB control in the civilian setting. The RCTC operates through a network of TB facilities, including 76 TB ambulatory centres, four regional centres, and 34 TB hospitals. The Ministry of Justice (MoJ) oversees TB control activities in correctional facilities, with technical support from the RCTC.^7^ The central prison hospital under the MoJ Tajikistan is the leading institute for TB control and treatment within the penitentiary system.^7^

The WHO recommends systematic TB screening in prisons, including screening when a prisoner enters the prison facility, annual screening, screening upon release, and symptomatic screening. The TB screening tools recommended by the WHO include 1) symptomatic screening for any TB symptoms, 2) chest radiography (CXR) for any abnormality, and 3) rapid molecular diagnostic tests adapted based on the local context.^8^ This study reports the yield from systematic TB screening conducted in four prisons and one pre-trial detention centre in Tajikistan, jointly conducted by Médecins Sans Frontières (MSF), the MoJ, and the MoH in Tajikistan.

## METHODS

### Study design and setting

This is a retrospective cross-sectional study aimed at identifying the yield of TB screening conducted in four prisons and one pre-trial detention centre around Dushanbe City, Tajikistan, between July 2022 and September 2023. Tajikistan is a landlocked country in Central Asia, with 93% of its territory covered by mountains. In 2023, the population was estimated to be 10.1 million,^9^ with an estimated 14,000 prisoners.^10^ The total prison population across the study sites was estimated to be around 6,500 individuals, representing 60% of the total prison population in Tajikistan.

### Screening procedures

A health promotion officer conducted a group health promotion session in a designated room inside the prison facility. The sessions covered TB infection, infection prevention and control measures, and the importance of TB screening in a confined setting. Verbal consent was obtained from each individual prisoner and prison staff member to undergo TB screening, and participation was voluntary.

Participants who consented were invited for a consultation by a trained TB specialist in a private room within the prison. A parallel screening consisting of symptomatic and CXR screenings was conducted, following a locally adapted screening algorithm (Figure) based on WHO recommendations.^11^ The participant’s weight, height, temperature, and medical history were assessed and recorded during the consultation. Self-reported symptomatic screening, including cough, fever, night sweats or weight loss, was included.

The availability and functionality of CXR varied across study sites. Some facilities had fully functional CXR capabilities throughout the study period, others had intermittent availability, and some lacked CXR capacity. Individuals with a positive screening, defined as the presence of any symptoms suggestive of TB, a risk factor for TB, or abnormalities in their CXR, underwent further evaluation with sputum testing using Xpert^®^ Ultra (Cepheid, Sunnyvale, CA, USA).

The sputum testing using Ultra was consistently performed across all study sites. Individuals suspected to have TB were instructed to provide a sputum sample taken in the early morning for Ultra testing. The sputum samples were transported to a laboratory supported by MSF for testing. In cases where GeneXpert results were unavailable, TB diagnosis was made clinically, with or without CXR findings, through consultation with a national TB consilium. All individuals with confirmed TB were further screened for HIV and given treatment. Symptomatic people with negative sputum test results were referred to the Penitentiary Health Service Unit (PHSU) for primary healthcare services.

Entry screening was conducted for individuals who had recently arrived at the pre-trial detention centre. Periodic screenings took place every 6 months for prison inmates and prison staff. Symptomatic and contact screenings were performed on an ad hoc basis for prison inmates referred by the PHSU.

### Data collection

Two register books were used for data collection during the screening process. The prison register book, accessible to the prison medical staff, recorded patient identifiers and other demographic and clinical characteristics. The MSF register book documented the same demographic and clinical characteristics without patient identifiers. A unique screening number was assigned to each patient to maintain confidentiality. This number was used to track individual prisoners with confirmed TB for further case management. The MSF register book was used to encode the data into a password-protected local database. Access to the database was restricted to the study investigators only.

### Data analysis

The baseline characteristics of the study population were presented as proportions for categorical variables and as median and interquartile ranges (IQRs) for continuous variables. The yield of TB screening was the proportion of individuals with confirmed TB out of the total number of screenings. Subgroup analyses assessed the relationship between TB diagnosis and CXR result status for participants who had undergone CXR and the relationship between TB diagnosis and CXR testing for symptomatic participants. The χ^2^ test was used to calculate the *P* value among categorical variables.

This study was approved by the MSF Independent Ethical Review Board and the MoJ in Tajikistan.

## RESULTS

A total of 7,223 active TB screenings were conducted among prison inmates and prison staff between July 2022 and September 2023, with 93% of the screenings being done in males. The median age of the participants was 34 years (IQR 27–43). Most of the screenings were entry screenings (*n* = 2,873, 40%) and periodic screenings (*n* = 4,208, 58%). Only 301 (4%) screenings were done in individuals with a body mass index (BMI) of less than 18.5 kg/m^2^ and 864 (12%) in people exhibiting at least one symptom of TB. Overall, 1,150 (16%) screenings were done in individuals who underwent CXR, and 1,086 (15%) underwent GeneXpert testing (Table 1).

**Table 1.** Baseline characteristics of the population screened (*n* = 7,223).

Characteristics	*n* (%)
Sex	
Female	482 (6.7)
Male	6741 (92.3)
Age, years, median [IQR]	34 [27–43]
Screening type	
Entry	2,873 (40)
Periodic	4,208 (58)
Contact	70 (1)
Staff	67 (1)
Exit	2 (0.01)
Unknown	3 (0.01)
Body mass index, kg/m^2^	
<18.5	301 (4)
18.5–24.9	3,899 (54)
>25	2,959 (41)
Unknown	64 (1)
Presence of any TB symptoms	
Yes	864 (12)
No	6359 (88)
Chest X-ray tested	
Yes	1150 (16)
No	6073 (84)
GeneXpert tested	
Yes	1,086 (15)
No	6,137 (85)

IQR = interquartile range.

In total, 31 TB cases were identified, comprising 17 cases of drug-susceptible TB, 8 cases of drug-resistant TB, and 6 clinically diagnosed cases. The screening yield was 0.43% (31/7,223 screenings). There was a statistically significant difference in the screening yield between screening in individuals who exhibited at least one TB symptom, at 3.4% (29 out of 864), and screening in those without any TB symptoms, at 0.03% (2/6,359; *P* < 0.001). Twenty-nine (94%) of the 31 positive screenings were in symptomatic individuals (Table 2).

**Table 2.** Screening outcome stratified by the presence or absence of any TB symptom for the study population.

Screening outcome	Presence of any TB symptoms		*P* value χ^2^ test
	Yes *n* (%)	No *n* (%)	
TB cases	29 (3.4)	2 (0.03)	<0.001
Clinically diagnosed TB, *n*	6	0	
Bacteriologically confirmed DR-TB, *n*	8	0	
Bacteriologically confirmed DS-TB, *n*	15	2	
Not TB	835 (96.6)	6357 (99.9)	
Total, *n*	864	6,359	

DS-TB = drug-susceptible TB; DR-TB = drug-resistant TB.

Subgroup analyses were conducted to assess the relationship between TB diagnosis and CXR result status for participants who had undergone CXR (Table 3), as well as the relationship between TB diagnosis and CXR testing for symptomatic individuals (Table 4). A significant difference in the rate of TB diagnosis was found between study participants with TB signs in their CXR at 21.1% (15/71 screenings) compared to those with abnormal CXR but without TB signs at 5.3% (1/19 screenings) and those with normal CXR at 0.3% (3/1,060 screenings; *P* < 0.001) (Table 3). Similarly, a significant difference in the rate of TB diagnosis was observed between symptomatic participants who had a CXR at 4.7% (19/408 screenings) and symptomatic participants who did not have CXR at 2.2% (10/456 screenings; *P* = 0.045) (Table 4).

**Table 3.** Relationship between being diagnosed with TB and the chest X-ray status among participants who had a chest X-ray.

Screening outcome	Presence of TB signs *n* (%)	Abnormal (no TB sign) *n* (%)	Normal *n* (%)	*P* value χ^2^ test
TB cases	15 (21.1)	1 (5.3)	3 (0.3)	<0.001
Not TB cases	56 (78.9)	18 (94.7)	1,057 (99.7)	
Total, *n*	71	19	1,060	

**Table 4.** Relationship between being diagnosed with TB and being tested for chest X-ray among symptomatic individuals screened.

Screening outcome	Had a chest X-ray		*P* value χ^2^ test
	Yes *n* (%)	No *n* (%)	
TB cases	19 (4.7)	10 (2.2)	0.045
Not TB cases	389 (95.3)	446 (97.8)	
Total, *n*	408	456	

Regarding the screening type, periodic screening accounted for 90% (28/31) of identified TB cases, while entry screening accounted for 10% (3/31). No TB cases were identified among prison staff, contacts of TB cases, or individuals undergoing exit screening.

## DISCUSSION

In this retrospective cross-sectional study in Tajikistan prisons, the yield of TB screening was 0.43% overall and 3.4% in people who exhibited at least one TB symptom. The screening yield of TB in our study is considerably lower than that of a previous study conducted by Winetsky et al. a decade ago in two prisons in Tajikistan, which reported a screening yield of 4.5% (59/1,317 prison inmates).^12^ Similarly, our finding is substantially lower than the screening yields reported in studies done in other high-burden countries in the region, such as Nepal (screening yield 1.4%; 6/434),^13^ India (1.9%; 14/738),^14^ Bangladesh (2.2%; 245/11,000),^15^ and Pakistan (4.25%; 44/1,027).^16^ These differences could partly be explained by variations in screening methods. Additionally, the overall decrease in the incidence of TB in Tajikistan from 108 cases/100,000 people in 2012 to 78 cases/100,000 people in 2022 could have contributed to the findings of our study.^1^

Nevertheless, some fundamental shortcomings in the TB screening activities conducted in our study may have contributed to the underestimation of the rate of TB in prison. First, only 16% of the study participants underwent CXR screening due to the unavailability of X-ray equipment in the prison facility. The WHO recommends parallel symptomatic and CXR screening to improve the sensitivity of TB screening. Studies have reported that the inclusion of CXR examinations increased the yield of TB screening activities.^17,18^

Second, the team only managed to collect early morning sputum samples for GeneXpert testing due to administrative challenges related to the transportation of sputum samples from the prison facilities to the laboratory. The WHO recommends collecting at least two sputum samples for TB diagnosis: one spot sample and one early-morning sample. Studies have suggested that differences in sputum collection methods greatly influence the diagnostic yield for TB.^19^ We acknowledge that these shortcomings could have contributed to the potential underestimation of the rate of TB in our study.

The burden of TB identified in our study was 429 cases/100,000 prison inmates, slightly lower than the rate reported by the WHO at 583 cases/100,000 inmates.^4^ This difference could be attributed to the underestimation of the rate of TB in our study. Nevertheless, in 2022, the estimated TB burden in Tajikistan's general population was 78 cases/100,000 population.^3^ This number is 5.5 times lower than the rate of TB identified in our study, implying that prison facilities remain a critical hotspot for spreading TB among inmates, and more effort should be made to enhance TB prevention and control in prison facilities.

Additionally, the observed difference in the rate of TB between symptomatic individuals who had a CXR test and symptomatic individuals who did not highlight the importance of CXR in active TB screening in a prison setting. This finding is consistent with WHO recommendations.^8^

## CONCLUSIONS

The study identified the rate of TB in the prison to be over five times higher than that in the general population in Tajikistan. Symptomatic individuals are more likely to be diagnosed with TB, and the use of CXR for active TB screening significantly increased the screening yield compared to symptomatic screening alone. We recommend expanding the capacity for CXR testing in prison facilities and reinforcing active TB screening upon entry, as well as periodic and symptomatic screening and exit screening, to enhance TB prevention and control in the prison setting in Tajikistan.

**Figure.**
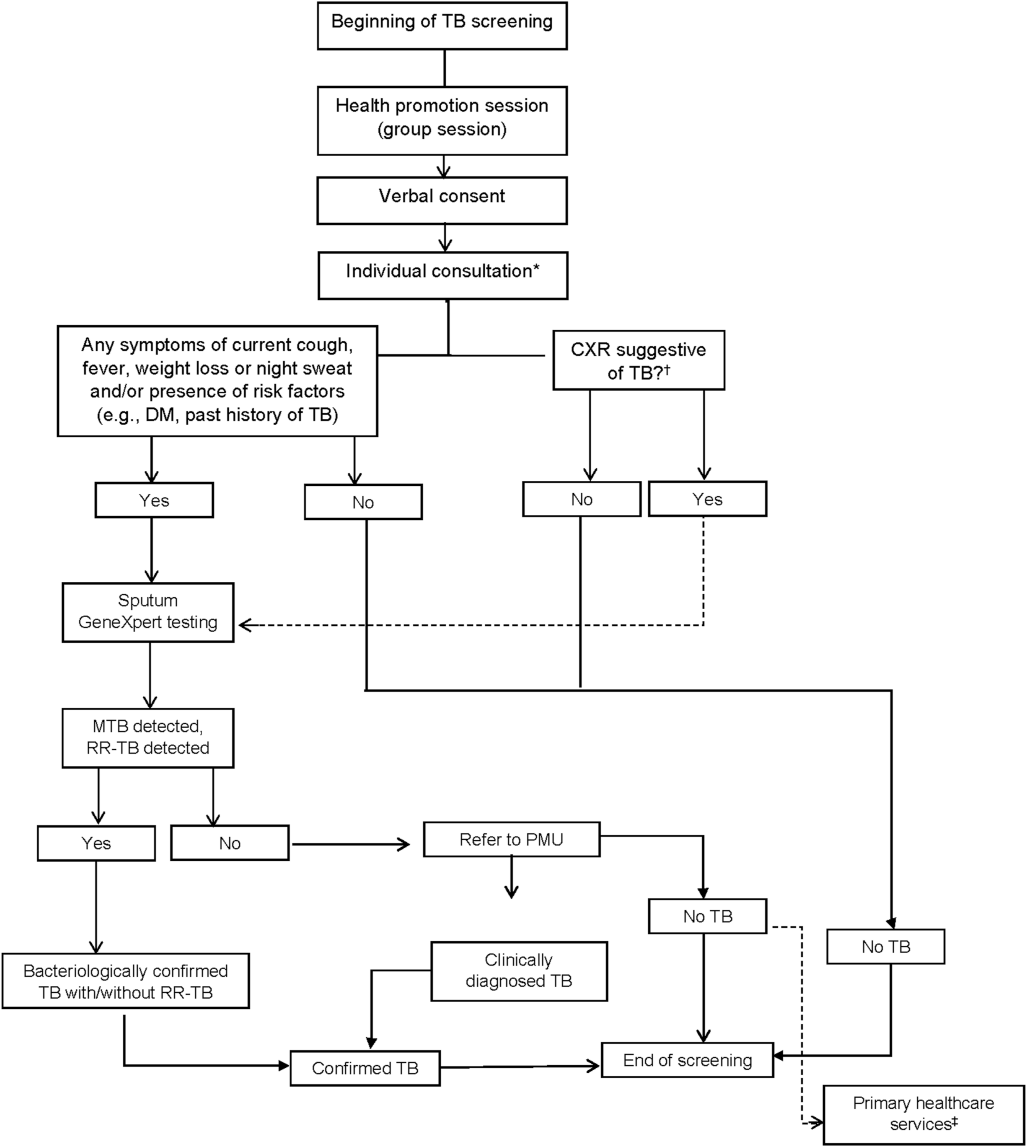
TB screening algorithm in prison. *Parallel symptomatic and CXR screening for all individuals. ^†^The availability of CXR varies among the prison facility. ^‡^The primary healthcare service is provided by the PMU. DM = diabetes mellitus; CXR = chest X-ray; MTB = *M. tuberculosis*; PMU = prison medical unit; RR-TB = rifampicin TB.

## References

[1] World Health Organization. Global tuberculosis report, 2023. Geneva, Switzerland: WHO, 2023.

[2] Baussano I, . Tuberculosis incidence in prisons: a systematic review. PLoS Med. 2010;7:e1000381. 21203587 10.1371/journal.pmed.1000381 PMC3006353

[3] World Health Organization. Country tuberculosis profile: Tajikistan 2022. Geneva, Switzerland: WHO, 2024.

[4] World Health Organization. Tuberculosis surveillance and monitoring in Europe 2022. Geneva, Switzerland: WHO, 2022.

[5] World Health Organization. Tajikistan is reforming primary health care to reach universal health coverage in 2023. Geneva, Switzerland: WHO, 2023.

[6] Central Asian Bureau for Analytical Reporting. Ten years for changes. Prison reform has begun in Tajikistan. Dushanbe, Tajikistan: CABAR, 2021.

[7] World Health Organization. Extensive review of tuberculosis prevention, control and care in Tajikistan. Geneva, Switzerland: WHO, 2013.

[8] World Health Organization. WHO consolidated guidelines on tuberculosis, module 2: screening, systematic screening for tuberculosis disease. Geneva, Switzerland: WHO, 2021. 33822560

[9] United Nations Population Fund. World population dashboard: Tajikistan. New York, NY, USA: UNFP, 2024.

[10] World Prison Brief, Institute for Crime & Justice Policy Research. London, UK. https://www.prisonstudies.org/country/tajikistan. Accessed April 2024.

[11] World Health Organization. WHO operational handbook on tuberculosis. Module 2: screening - systematic screening for tuberculosis disease. Geneva, Switzerland: WHO, 2021. 33822560

[12] Winetsky DE, . Prevalence, risk factors and social context of active pulmonary tuberculosis among prison inmates in Tajikistan. PLoS One. 2014;9:e86046. 24465861 10.1371/journal.pone.0086046 PMC3896449

[13] Shrestha G, . Pulmonary tuberculosis among male inmates in the largest prison of Eastern Nepal. Tuberc Res Treat. 2019;2019:3176167. 31687207 10.1155/2019/3176167 PMC6803721

[14] Bhatnagar T, . Intensified tuberculosis and HIV surveillance in a prison in Northeast India: implementation research. PLoS One. 2019;14:e0219988. 31356606 10.1371/journal.pone.0219988 PMC6662996

[15] Banu S, . Pulmonary tuberculosis and drug resistance in Dhaka central jail, the largest prison in Bangladesh. PLoS One. 2010;5:e10759. 20505826 10.1371/journal.pone.0010759 PMC2874010

[16] Shah SA, . Screening of jail inmates for HIV and tuberculosis. Pak J Med Health Sci. 2013;7:172–175.

[17] Velen K, . Digital chest X-ray with computer-aided detection for tuberculosis screening within correctional facilities. Ann Am Thorac Soc. 2022;19:1313–1319. 34914539 10.1513/AnnalsATS.202103-380OC

[18] Nalunjogi J, . Accuracy and incremental yield of the chest X-ray in screening for tuberculosis in Uganda: a cross-sectional study. Tuberc Res Treat. 2021;2021:6622809. 33828862 10.1155/2021/6622809 PMC8004368

[19] Datta S, . Comparison of sputum collection methods for tuberculosis diagnosis: a systematic review and pairwise and network meta-analysis. Lancet Glob Health. 2017;5:e760–e771. 28625793 10.1016/S2214-109X(17)30201-2 PMC5567202

